# Coupled CFD-FEM analysis of the damage causes of the retention bunker: a case study at hard coal mine

**DOI:** 10.1038/s41598-024-65034-z

**Published:** 2024-06-20

**Authors:** Tomasz Janoszek, Marek Rotkegel

**Affiliations:** Department of Extraction Technologies, Rockburst and Risk Assessment, Central Mining Institute – National Research Institute, 40-166, Katowice, Poland

**Keywords:** Retention bunker, Hard coal mine, Coal-water mixture, FEM, CFD, Numerical model, Engineering, Mechanical engineering

## Abstract

Underground coal storage bunkers serve as crucial infrastructural components in the coal mining industry, providing secure and accessible locations for the storage of mined coal. The interaction between stored coal and underground water in coal storage bunkers indeed poses significant challenges due to the unpredictable nature of the resulting coal-water mixture. This phenomenon is particularly prevalent in coal mines operating under water hazards, where groundwater infiltration into storage areas can lead to the formation of coal-water mixtures, altering the physical properties of the stored coal. The interaction between coal and water can result in the formation of coal-water mixtures (hydromixture), which exhibit complex rheological properties. These mixtures may vary in viscosity, density, and particle size distribution, making their behavior difficult to predict. Underground water may exert hydrostatic pressure on the stored coal, influencing its mechanical behavior and compaction properties. Changes in pressure can result in coal compaction or expansion, affecting bunker stability and the integrity of surrounding rock strata. The main goal of the paper was to determine the values of pressure field variations exerted by the flowing hydromixture within underground coal storage bunkers. This objective reflects a critical aspect of understanding the dynamic behavior of coal-water mixtures (hydromixture) under varying conditions, particularly in environments where water hazards pose significant challenges to storage and operational stability. The paper utilized computational fluid dynamics (CFD) methods to examine the changes in pressure within underground coal storage bunkers induced by the flow of coal-water mixtures. The examination of damage to an underground coal storage bunker due to stress distribution was conducted using the finite element method (FEM). This computational technique is widely utilized in engineering and structural analysis to model complex systems and predict the behavior of materials under various loading conditions The results of the CFD numerical simulation were compared with the mathematical models.

## Introduction

Retention bunkers serve as crucial components in the mining sector, primarily used for storing and controlling the flow of mined coal to the surface. Underground coal storage bunkers serve as large containers capable of holding significant quantities of coal, providing flexibility to accommodate fluctuations in production and demand. These structures play a crucial role in ensuring a continuous and reliable supply of coal to surface operations while allowing for efficient handling and transportation^1^. Underground coal storage bunkers are designed to hold massive amounts of coal, providing a buffer to accommodate increases in production or disruptions in transportation. Internal loads from stored coal within underground storage bunkers can be estimated based on industry standards or guidelines specific to coal storage facilities described in^2,3^. Standards for shaft enclosures can serve as valuable references. To assess the operation of the retention bunker construction and the state of its stress, it is critical to know the size of the loads that the bunker is subjected to. During filling and emptying operations, the movement of coal within the bunker induces flow dynamics, including bulk flow, compaction, segregation, and bridging. These flow phenomena can lead to non-uniform loading patterns, localized stress concentrations, and potential instabilities within the bunker structure. Understanding and managing these dynamic flow processes are essential for ensuring the structural integrity and operational safety of the bunker. The issue of internal loads exerted by stored coal on the construction of retention bunkers remains a complex and unresolved challenge. The authors in the study^4^ introduce a stochastic model designed to determine optimal bunker placements and capacities within a system featuring a single production section and a series of interconnected conveyors The paper^5^ calculates the grain size distribution of the carried copper ore based on photos taken over the main haulage conveyor, which feeds mined material to the underground bunker located at the skip-filling station of the shaft. The authors of paper^2^ compiled illustrations depicting typical bunker damage and outlined research techniques for evaluating their technical condition. An important and practical challenge in coal mine production is selecting the optimal location for the coal bunker. Due to the intricate interplay between influencing variables and optimization objectives, linear programming often results in a local rather than a global optimization solution. To address this issue and determine the optimal position for the coal bunker within the coal mine transportation system, the authors of research^6^ propose a combination of a nonlinear programming model and the particle swarm optimization (PSO) technique.Equipment used in retention bunkers can experience abrasive wear due to the handling of granular materials such as rocks, pebbles, and sand, leading to potential local failure. Understanding how granular material flows during transport in a granular material handling system is a critical characteristic to consider in designing and operating retention bunkers effectively. In the study^7^, the structure of the tipper body is modeled using the finite element approach, while the granular material is modeled using the discrete element method. This combination of modeling techniques allows for a comprehensive analysis of both the structural behavior of the tipper body and the flow dynamics of the granular material it handles.One significant issue in underground coal mines is the severe damage caused by floor heave in the coal-filled chamber of a vertical coal bunker. In conventional vertical coal bunker systems, the entire weight of the bunker is typically supported by a coal-feeder chamber (CFC). However, maintaining CFCs within soft, expanding floor rock poses a significant challenge for underground coal mines. Additionally, the primary zone of deformation for the entire rock mass surrounding the bunker occurs within the coal seam section that extends into the interior of the bunker. For the construction of vertical bunkers without CFCs in coal mines with soft, expanding floor rocks, the authors of the paper^8–11^ provide valuable references. In the paper^12^, a mechanism for preventing coal bunker blocking was described. This mechanism aims to prevent coal bridging and ensure effective feeding of coal into the bunker. The paper^13,14^ investigated the coal storage behavior of bunker construction. The authors presented a case study involving a stacker chute system that transfers coal from one receiving conveyor to one outgoing (boom) conveyor to demonstrate the application of two techniques in a real-life high-throughput installation. The study utilized site observations and discrete element method (DEM) simulations to evaluate the effectiveness of traditional and modified continuum method approaches. Furthermore, the paper includes a sensitivity analysis of the modeling parameters. The stability of the retention bunker under excavation slope conditions, considering deformation with and without reinforcement were tested in^15^. The modeling results indicate that the stress field redistributes during excavation and reinforcing, effectively preventing horizontal slope displacement. However, it is noted that the soil at the base of the bunker needs to be strongly compacted and supported by piles. To predict deformation trends, design reinforcement schemes, reduce excessive heave at the base of excavation, minimize horizontal displacement of the excavation slope, and ensure project safety, the stability of the bunker was simulated using the finite element method. The main finding of the research was that abrasive processes lead to corrosion and wear of steel components. In study^16^, tests were conducted to identify areas of the hopper surface that were more damaged and to determine how grain shape affected the bunker’s performance. The relative wear on hoppers caused by friction during operation was assessed using a discrete element technique. Additionally, the wear and tear on steel elements of bunkers operated in underground copper ore mines due to abrasive processes were analyzed in paper^17^. The bunker elements’ linings were found to undergo severe abrasive wear. Among various parameters influencing the abrasive wear of the examined bin elements, the varied physical and mechanical properties of the copper ore were identified as the most significant. Additionally, the safety construction of the coal bunker, focusing on the thickness of the safety aquifer in the bunker roof and the water drainage ability of the aquifer in the sandstone of the roof, was studied in^18^.

The analysis of available literature highlights two main areas of concern regarding underground coal storage bunkers: abrasion processes associated with material transport and the influence of geological and hydrogeological conditions on bunker deformation and stability. These issues have been addressed using stochastic methods and the discrete element method (DEM) numerical method.

The strength and functionality of the retention bunker outlet closure devices are crucial for the correct and trouble-free operation of transporting excavated material to the surface The safety of crews operating the bunker during the dispensing of excavated material, especially underground coal, is paramount. Control measures for the dispensing process from the bunker to the conveyor belt are crucial for maintaining a safe working environment. Engineering knowledge combined with regulations forms the foundation for designing appropriate retention bunker enclosures, surface linings, and strutting elements to mitigate abrasion and ensure structural integrity. The design of the retention bunker outlet also requires a proper approach. Designing a functional and reliable mechanism for metering the spoil and closing the outlet from the bunker is crucial for efficient material handling and operational safety. Additionally, using appropriately strong components, such as plating sheets, stiffeners, screw connections, and other structural elements, ensures the durability and integrity of the outlet structure. At the design stage, engineers must carefully consider the anticipated operating conditions of the device to ensure its effectiveness, reliability, and safety. In this design issue, they are strongly related to the properties of the material stored in the bunker—the underground coal. In the case of the storage of bulk materials, including the underground rock and coal, an important parameter is the moisture content of the material and its accumulation of water, which significantly affects the behaviour of the excavated material. The increased water content of the stored material creates a hydromixture, which has a completely different effect on the bunker outlet closure devices. The load in hydrostatic or hydrodynamic forms affects all the walls of the outlet, and its value is greater than the dry and loose-grained excavated materials. This can lead to exceeding the strength of the various components of the bunker structure and damaging them. Consequently, this leads to the uncontrolled discharge of unreliable excavated material in the form of hydromixture and filling the chamber space under the retention bunker. Examples of such incidents include the recent accidents that occurred at the Ziemowit^19^ and Bogdanka^20^ hard coal mines.

The incident at the Piast-Ziemowit hard coal mine on February 19, 2021, involved the activation of a fire extinguishing device in the Z-3 retention bunker. The water flowing into the excavated material stored in the reservoir bunker, causing coal and rock suspended in water to enter the roadway. Seven workers were tasked with addressing the failure, but during their work, another hydromixture of coal and rock entered the coal mine roadway from the underground bunker. The hydromixture engulfed the working employees, resulting in six of them escaping from the roadway, while one worker was buried and tragically died^19^. The hydromixture filled the coal mine roadway nearly halfway to its height.At LW Bogdanka S.A., on June 8, 2022, there was an outflow of coal and rock suspended in water from the 5fB retention bunker to the coal mine roadway at a depth of 920 m. The retention bunker collected excavated materials from one longwall and three coal mine roadways. At the time of the accident, there were four miners in the roadway. Due to the uncontrolled outflow of coal and rock suspended in water, one miner was partially buried, and tragically, a second miner was completely buried and died as a result^20^. The outflowing mixture, approximately 280 m^3^ in volume, filled the mine roadway to more than half its height, reaching a maximum of 2.8 m.

The reliability of retention bunker devices is paramount as it directly impacts the safety of personnel working with and around them. Incidents involving retention bunkers can lead to serious consequences, including injuries and fatalities. Therefore, ensuring the reliability of these devices through robust design, regular maintenance, and adherence to safety protocols is crucial for protecting the well-being of workers and maintaining safe operations in mining environments.

The article analysis the causes of damage to retention bunker construction elements under typical operating conditions. It employs numerical methods to replicate conditions and investigate the factors contributing to bunker damage. Computational Fluid Dynamics (CFD) solver, specifically SolidWorks Flow Simulation, was utilized to analyze pressure distribution within the bunker. The results from CFD simulations were used to assess mechanical parameters of the retention bunker model using the Finite Element Method (FEM) solver in Solidworks 2018 Simulation. To achieve this, a 3D model of the underground bunker was reconstructed, initial and boundary conditions were defined, and simulation results were meticulously analyzed.

## Materials and methods

### Retention bunker in-situ analysis

**Figure 1:**
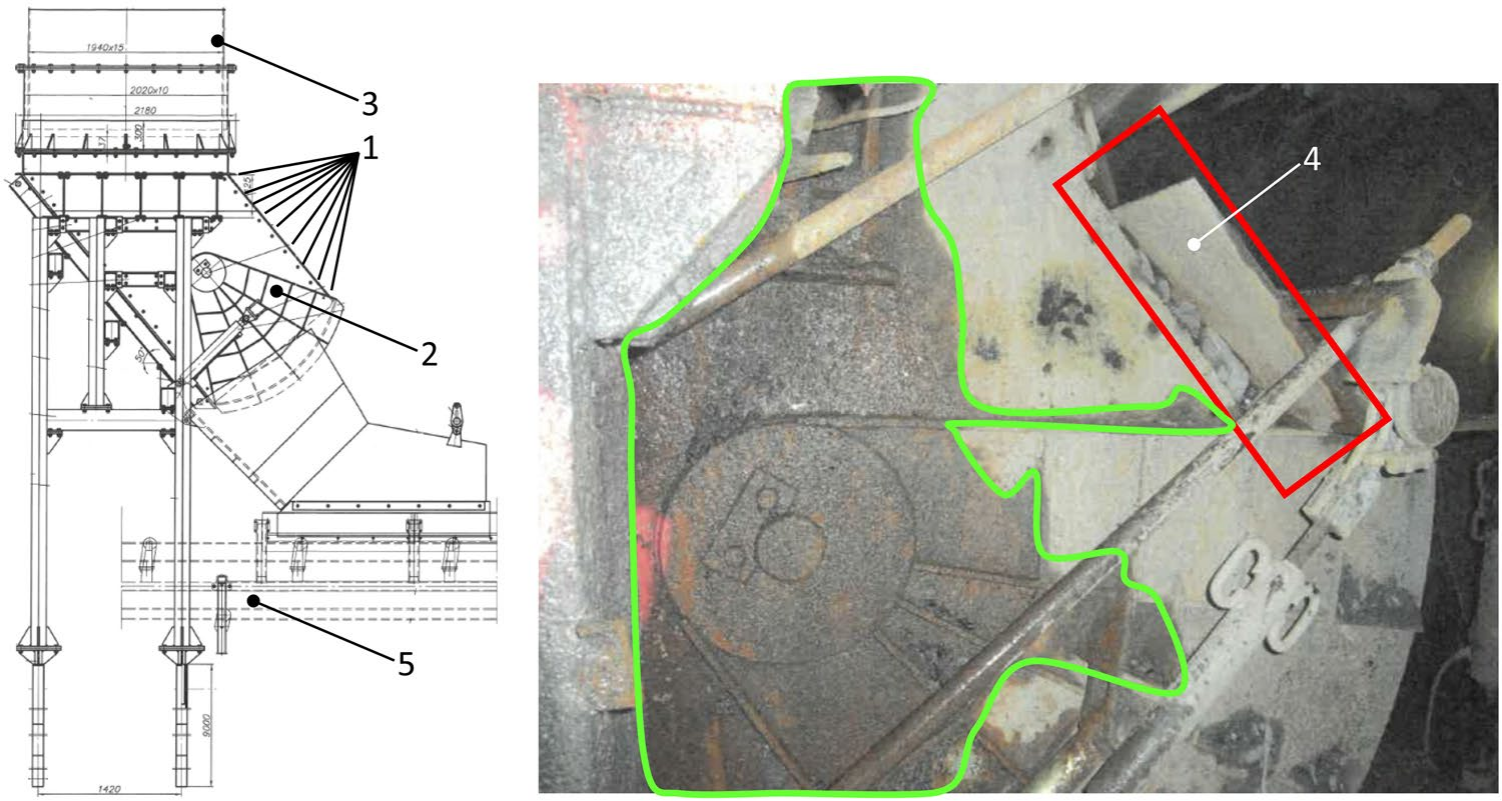
Retention bunker during the in-situ inspection of damages: 1—screw connection, 2—receiving bin, 3—vertical tube, 4—damaged upper shield, 5—conveyor belt.

Figure 1 depicts the underground coal bunker in its in-situ conditions with the observed damage. Specifically, the screw connection (identified as number 1 in Fig. 1) has become disconnected, leading to a leak in one of the bunker’s shields (identified as number 4 in Fig. 1). Consequently, all stored material within the retention bunker has spilled into the underground excavation, disturbing the proper transport of coal via the conveyor belt.

During the in-situ inspection, moisture stains were observed on the outer surface of the bunker. These moisture stains are depicted in Fig. 1 as a green line. The presence of these stains indicates that natural or process water has infiltrated the coal stored within the retention bunker. This water-coal interaction has resulted in the formation of a hydromixture, altering the physical properties of the coal from a static solid to a dynamic fluid. Consequently, this change has exacerbated the stability issues of the underground bunker structure.

### Retention bunker under the load

**Figure 2:**
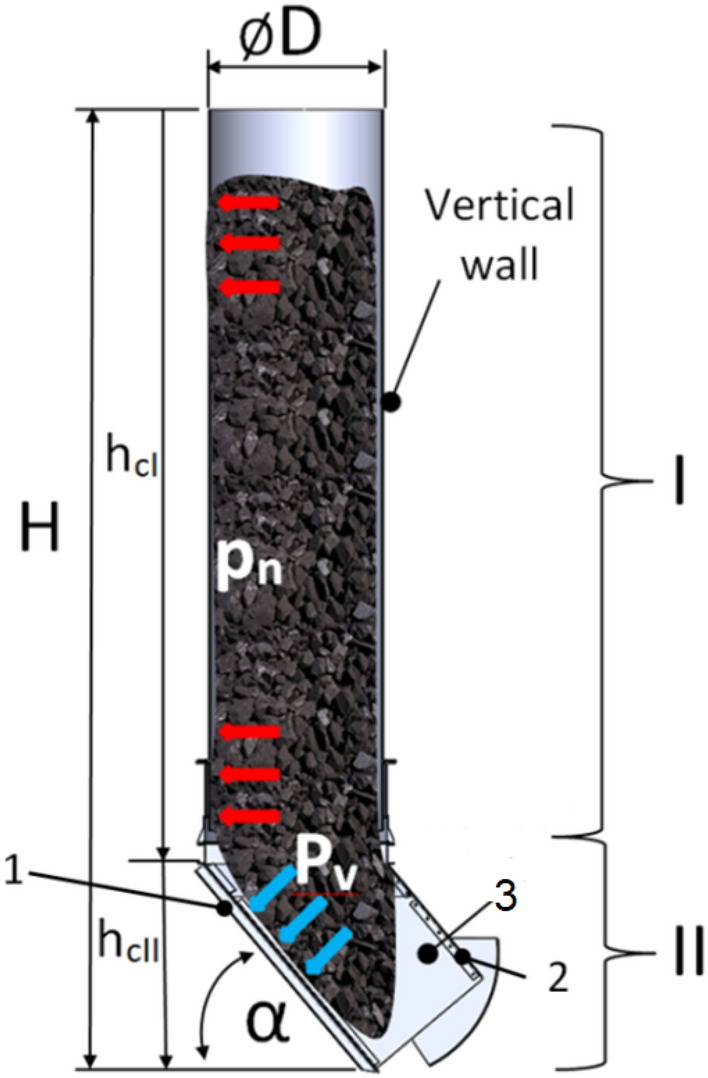
Dimensions and determination of static pressures in retention bunker: 1—lower shield, 2—upper shield, 3—receiving bin (hopper).

Due to its purpose, a retention bunker generates mass forces as a result of the stored solid material. These mass forces within the gravitational field produce static pressure, which increases with the depth of the bunker. The magnitude of the mass forces is influenced by the density of the material. Figure 2 illustrates an example of the static pressure distribution along the underground bunker during the storage of solid material. The geometry of the bunker is divided into two zones based on the pressure distribution. Zone I represents the area where the hydraulic pressure (pn) is directed normally towards the vertical wall of the bunker. Zone II depicts the distribution of hydraulic pressure (pv) oriented vertically towards the wall of the receiving bin (Fig. 2-1, 2).

The bunker is loaded with a solid material along the vertical wall (tube) and along the receiving bin shield, as can be observed from the arrangement of forces illustrated in Fig. 2. However, because the upper shield of the bunker (Fig. 2-2,1-4) does not have contact with the accumulated material, the additional reinforcement of the upper shield to protect against potential additional loads was not considered. Therefore, it is possible that the change from a solid material (coal) to a two-phase hydromixture (coal and rock suspended in water) may have caused damage to the upper shield (Fig. 2-2).

The value of the hydraulic pressure along zone I due to the stored solid material can be estimated based on the equation:

1 $${p}_{n}=\rho \cdot g\cdot {\Delta h}_{cI}$$

The value of the hydraulic pressure (p_v_) along zone II can be estimated based on the equation:

2 $${p}_{v}=\rho \cdot g\cdot \frac{{\Delta h}_{cII}}{sin\alpha }$$

where: ${p}_{n}$—pressure along the vertical wall of the bunker, N m^−2^. ${p}_{v}$—pressure along the receiving bin of the bunker, N m^−2^. $\rho$—density, kg m^−3^. $g$—gravity, m s^−2^. ${\Delta h}_{cI}$—depth increment of the vertical wall (tube) of the bunker, m. ${\Delta h}_{cII}$—depth increment of the receiving bin of the bunker, m. $D$—diameter, m. $\alpha$—inclination angle of the receiving bin (hopper). The force distribution (F) along the retention bunker can be estimated based on the equation:

3 $$F=\Delta H\cdot \pi \cdot {(\frac{D}{2})}^{2}\cdot \rho \cdot g$$

where: $F$—force, N. $\rho$—density, kg m^−3^. $g$—gravity, m s^−2^. $\Delta H$—depth increment, m

**Figure 3:**
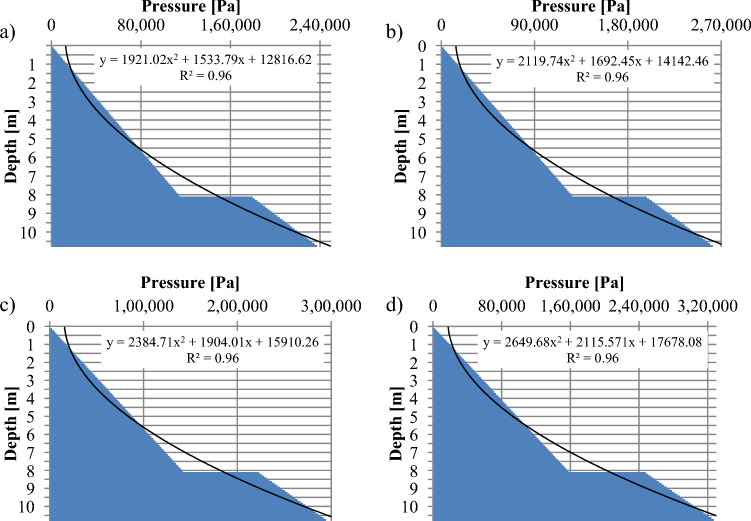
Static pressure distribution depending on the depth and the density: (**a**)—ρ = 1450 kgm^−3^, (**b**)—ρ = 1600 kgm^−3^, (**c**)—ρ = 1800 kgm^−3^, (**d**)—ρ = 2000 kgm^−3^.

An essential characteristic of coal is density. The investigations in^21^ demonstrated that increased density can be achieved by an agglomeration process when there is an excess of moisture. Small particles of coal combine into larger ones and take on a new form of structure, characterised by individual properties different from those of the original particles. The agglomeration process uses intermediate media that directly affect the rate of agglomerate formation. These include, for example, water^22^. Depending on the particle size, impurities, and moisture, the bulk density of the hard coal can change from 700 to 1100 kgm^−3^^23^. For the model study, it was assumed that the density of the hydromixture varied within the range of 1450–2000 kgm^−3^. This range was chosen to encompass a realistic spectrum of densities that the hydromixture could potentially exhibit under different operating conditions. By considering this range, the study aimed to capture the potential variations in material properties and their effects on bunker performance and stability. Studies referenced in the paper^24–26^ This coefficient plays a significant role in determining the resistance to sliding or movement between solid surfaces in contact with each other. In the case of water inflow, slippage occurs at the boundary between grain surfaces, initiating the process of movement between contacting surfaces. Assuming a coal density of 1100 kgm^−3^, the hydromixture density equal to 1450 kgm^−3^, the volume fraction of coal is 0.75 and the water is 0.25; for 1600 kgm^−3^, the volume fraction of coal is 0.69 and 0.31; for 1800 kgm^−3^, the volume fraction of coal is 0.61 and the water is 0.39; and for density 2000 kgm^−3^, the volume fraction of coal is 0.55 and the water is 0.45. Relationships (1) and (2) were interpreted in Fig. 3 in function of the hydromixture density and the bunker depth. The real geometrical parameters of the retention bunker were adopted in the calculations as shown in Fig. 2: the bunker diameter (D) is 1.91 m, the inclination angle of the receiving bin is 40°, the total depth (H) of the bunker is 10.7 m, where the depth of zone I is 8.1 m.

In Fig. 3, the following observations can be made regarding the relationship between material density and maximum pressure within the retention bunker:

- For a density of 1450 kg/m^3^, the maximum pressure is approximately 240 kPa.
- For a density of 1600 kg/m^3^, the maximum pressure is approximately 270 kPa.
- When the material density reaches 1800 kg/m^3^, the maximum pressure recorded is approximately 300 kPa.
- For a density of 2000 kg/m^3^, the maximum pressure is approximately 320 kPa

**Figure 4:**
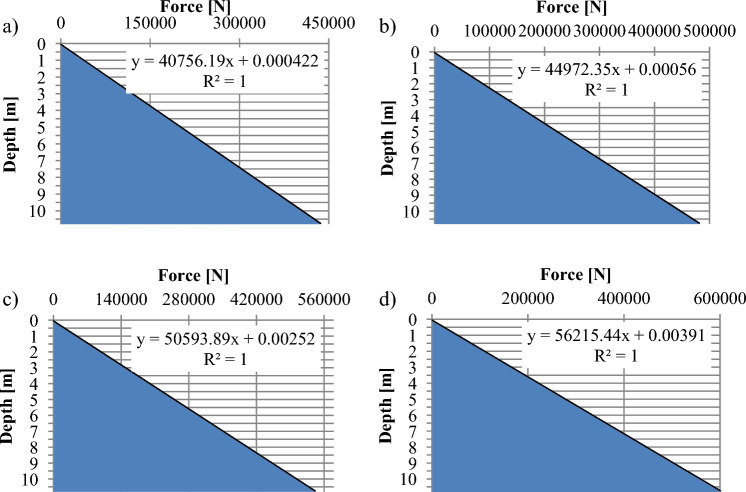
Force distribution depending on the depth and the density: (**a**)—ρ = 1450 kgm^−3^, (**b**)—ρ = 1600 kgm^−3^, (**c**)—ρ = 1800 kgm^−3^, (**d**)—ρ = 2000 kgm^−3^.

In the case of a change in bunker depth, it is possible to observe a linear increase in static pressure up to a certain depth. This linear increase occurs as a result of the gravitational forces acting on the material stored within the bunker, causing the pressure to incrementally rise with depth. Below the 8-m level, a significant increase in static pressure can indeed be observed, amounting to approximately 60%. This increase is primarily attributed to the change in the inclination angle (α) of the receiving bin. The results of the calculations indicate that depth and density are significant variables that influence the load on a retention bunker. In Fig. 4, the distribution of forces along the retention bunker illustrates that the force value is indeed dependent on both the density and height of the material stored within the bunker.

The maximum value of the force distribution in Fig. 4 varies as follows:

- For a density of 1450 kg/m^3^, the maximum force is 432 kN (Fig. 4-a).
- For a density of 1600 kg/m^3^, the maximum force increases to 472 kN (Fig. 4-b).
- When the density reaches 1800 kg/m^3^, the maximum force further increases to 526 kN (Fig. 4-c).
- Finally, for a density of 2000 kg/m^3^, the maximum force reaches 590 kN (Fig. 4-d).

Figures 3 and 4 indicate that the shield (Fig. 2-1) of the receiving bin is subjected to significant loads. The damage occurred as a result of dynamic mass forces associated with the formation of a hydromixture consisting of water and coal. Equations (1), (2), and (3) represent a static model that describes the behavior of the retention bunker under static conditions. These equations do not account for dynamic phenomena such as the formation of hydromixtures, fluid flow, or other transient effects.The hydromixture, characterized by its fluidity and instability, poses a potential threat due to the changing load it imposes on the retention bunker.To estimate the dynamic influence on the strength of the underground bunker construction, Computational Fluid Dynamics (CFD) methods were utilized^22^. The results obtained from the Computational Fluid Dynamics (CFD) simulations provided valuable insights into the dynamic behavior of the hydromixture within the bunker. These insights were then used to predict the stress distribution in the bunker’s elements using the Finite Element Method (FEM).

### Numerical analysis

In order to calculate the values of pressure field variations in the volume limited by the retention bunker construction, model tests were carried out.

#### Numerical grid

The Navier–Stokes equations, which are formulations of the laws of conservation of mass, momentum, and energy, are solved in fluid regions of the retention bunker model, namely^22,27^:

- Conservation of mass:

4 $$\frac{\partial \rho }{\partial t}+\frac{\partial (\rho {u}_{i})}{\partial {x}_{i}}=0$$

- Conservation of momentum:

5 $$\frac{\partial (\rho {u}_{i})}{\partial t}+\frac{\partial (\rho {u}_{i}{u}_{j})}{\partial {x}_{j}}+\frac{\partial P}{\partial {x}_{i}}=\frac{\partial }{\partial {x}_{j}}\left({\tau }_{ij}+{\tau }_{ij}^{R}\right)+{S}_{i}$$

- Conservation of energy:

6 $$\frac{\partial \rho H}{\partial t}+\frac{\partial \rho {u}_{i}H}{\partial {x}_{j}}=\frac{\partial }{\partial {x}_{j}}\left({u}_{j}{(\tau }_{ij}+{\tau }_{ij}^{R})\right)+\frac{\partial p}{\partial t}-{\tau }_{ij}^{R}\frac{\partial {u}_{i}}{\partial {x}_{j}}+\rho \varepsilon +{S}_{i}{u}_{i}+{Q}_{H}$$

7 $$H=h+\frac{{u}^{2}}{2}$$

where: $\rho$—density, [kg m^3^]. *t*—time, [s]. *u*_*i*_*, u*_*j*_—axial velocity, [m s^−1^]. *x*_*i*_, *x*_*j*_—axial coordinate, [m]. ${\tau }_{ij}$—stress tension, ${\tau }_{ij}=\mu {s}_{ij}$; ${\tau }_{ij}^{R}={\mu }_{t}{s}_{ij}-\frac{2}{3}\rho k{\delta }_{ij}$. *p*—static pressure, [Pa]. *S*_*i*_—source of momentum, *H*—total enthalpy, [J·(kg K)^−1^]. *h*—enthalpy, [J·(kg K)^−1^]. *Q*_*H*_—heat flux, [W m^−2^].

**Figure 5:**
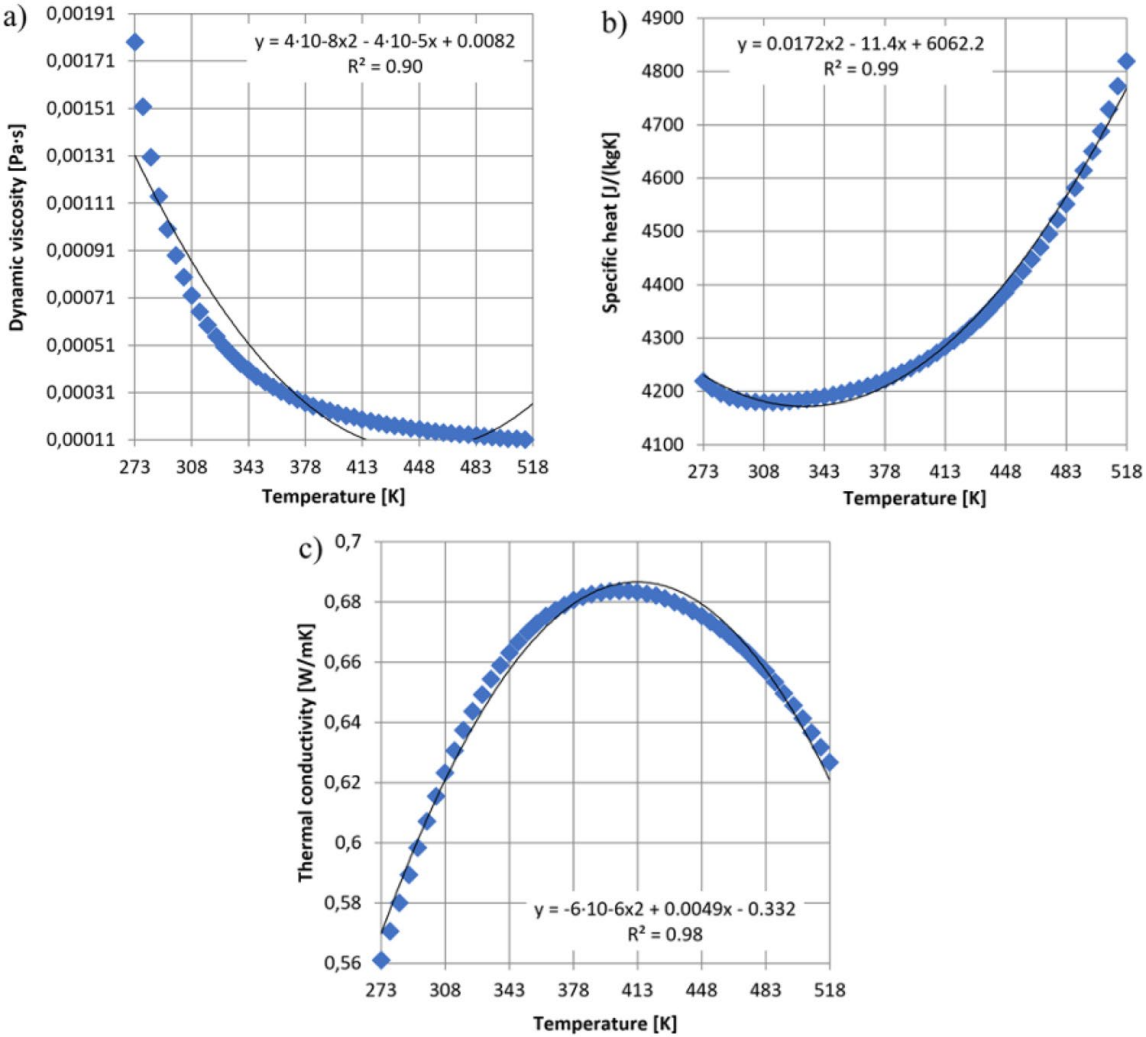
Adopted parameters of the hydromixture depending on the temperature: (**a**)—dynamic viscosity, (**b**)—specific heat, (**c**)—thermal conductivity.

These equations are completed with equations describing the fluid’s state as well as empirical relationships between temperature and the fluid’s density, viscosity, and thermal conductivity. During the numerical simulations, it was assumed that the inflow of fluid (water) into the space of an underground bunker filled with excavated material (coal) resulted in a loss of stability. This loss of stability led to a dynamic outflow of coal and water in the form of a hydromixture towards the outlet of the retention bunker. This scenario reflects the potential conditions where the inflow of water into the bunker can destabilize the stored material, leading to fluidization and the formation of a hydromixture. In Fig. 5, the variation of the physical parameters of the hydromixture, including dynamic viscosity (Fig. 5a), specific heat (Fig. 5b), and heat transfer coefficient (Fig. 5c), is characterized by the corresponding graphs as a function of temperature.

The transport equations for the turbulent kinetic energy and its dissipation rate were employed using the k-ε model. The laminar, turbulent and transitional flows of homogeneous fluids are described by the following turbulence conservation laws in the modified k-turbulence model with damping functions proposed by^28^:

- For turbulence kinetic energy:

8 $$\frac{\partial \rho k}{\partial t}+\frac{\partial \rho {ku}_{i}}{\partial {x}_{i}}=\frac{\partial }{\partial {x}_{i}}\left(\left(\mu +\frac{{\mu }_{t}}{{\sigma }_{k}}\right)\frac{\partial k}{\partial {x}_{i}}\right)+{\tau }_{ij}^{R}\frac{\partial {u}_{i}}{\partial {x}_{j}}-\rho \varepsilon +{u}_{t}{P}_{B}$$

- For dissipation energy:

9 $$\frac{\partial \rho \varepsilon }{\partial t}+\frac{\partial \rho {\varepsilon u}_{i}}{\partial {x}_{i}}=\frac{\partial }{\partial {x}_{i}}\left(\left(\mu +\frac{{\mu }_{t}}{{\sigma }_{\varepsilon }}\right)\frac{\partial \varepsilon }{\partial {x}_{i}}\right)+{C}_{\varepsilon 1}\frac{\varepsilon }{k}\left({{f}_{1}\tau }_{ij}^{R}\frac{\partial {u}_{i}}{\partial {x}_{j}}+{C}_{B}{\mu }_{t}{P}_{B}\right)-{f}_{2}{C}_{\varepsilon 2}\frac{\rho {\varepsilon }^{2}}{k}$$

where: *C*_*ε1*_—empirical constant, C_ε1_ = 1.44, *C*_*ε2*_—empirical constant, C_ε2_ = 1.92, *C*_*µ*_—empirical constant, C_µ_ = 0.09, *k*—velocity fluctuation (turbulence) kinetic energy [m^2^ s^−2^], *P*_*B*_—local vorticity fluctuation production, *ε—*turbulence kinetic energy dissipation rate [m^2^ s^−3^], *μ*_*t*_—turbulent viscosity [Pa s], *σ*_*k*_—turbulent Prandtl number σ_k_ = 1.0, *σ*_*ε*_—turbulent Prandtl number σ_ε_ = 1.3, *σ*_*B*_—turbulent Prandtl number σ_B_ = 0.9, *µ*—fluid dynamic viscosity [Pa s], *f*_*1*_, *f*_*2*_—empirical constant.

The Finite Elements Method (FEM) approach was used to do calculations in a SolidWorks simulation module. Computer programmers utilize internal forces and displacements, along with computational methods based on the finite element method (FEM), to automatically calculate the Huber-Mises-Hencky reduced stress. The von Mises-Hencky theory, also known as the Maximum distortion energy theory or the Shear-energy theory, forms the basis for the maximum von Mises stress criterion. The von Mises stress can be expressed in relation to the major stresses σ_1_, σ_2_, and σ_3_ as follows^29^:

10 $${\sigma }_{von Mises}=\{[{\frac{\left[{\left({\sigma }_{1}-{\sigma }_{2}\right)}^{2}+{\left({\sigma }_{2}-{\sigma }_{3}\right)}^{2}+{\left({\sigma }_{1}-{\sigma }_{3}\right)}^{2}\right]}{2}]\}}^{0.5}$$

According to the theory, a ductile material begins to yield or deform plastically when the stress limit is reached by the von Mises stress. The stress limit is typically determined using the yield strength.

The following boundary conditions were considered:

1. In the case of the CFD solver:

- Dynamic viscosity of hydromixture depending on the temperature: µ(T) = 4e^−8^T^2^−4e−5 T + 0.0082 [Pa s] (Fig. 5a),
- Specific heat of hydromixture depending on the temperature: Cp(T) = 0.0172T^2^–11.4 T + 6062.2 [J kg^−1^ K^−1^] (Fig. 5b),
- Thermal conductive of hydromixture depending on the temperature: λ(T) = −6e^−6^T^2^ + 0.005 T−0.332 [W m^−1^ K^−1^] (Fig. 5c),
- Temperature: T = 298.15 [K] (25[°C]),
- Density: from 1450 to 2000 kg m^−3^,
- Turbulent model: k-ε,
- Time of calculations: 3 s,
- Gravity: 9.81 [m s^−2^].

2. In the case of the FEM solver:

- Type of FEM analysis: linear static,
- Coefficient of elasticity: 210,000 [N mm^−2^],
- Poisson’s ratio: 0.28,
- Shear stress coefficient: 79,000 [N mm^−2^],
- Density: 7800 [kg m^−3^],
- Tensile strength: 360 [N mm^−2^],
- Yield strength: 235 [N mm^−2^],
- Thermal conductivity coefficient: 14 [W (m K)^−1^],
- Specific heat: 440 [J (kg K)^−1^].

#### Numerical grid

In order to simplify the issue, it was assumed that the cross-section of the retention bunker is constant throughout the analysed vertical section. In reality, it is variable. In the upper part, the bunker has a cylindrical cross-section with a diameter of about 1.91 m, which changes to a square cross-section in the lower part of the bunker (receiving bin). This approach makes it possible to disregard possible turbulence generated at the points where the cross-section changes. Despite the simplifications adopted, the model allows us to learn about the phenomena occurring in a reservoir filled with unreliable spoil.

**Figure 6:**
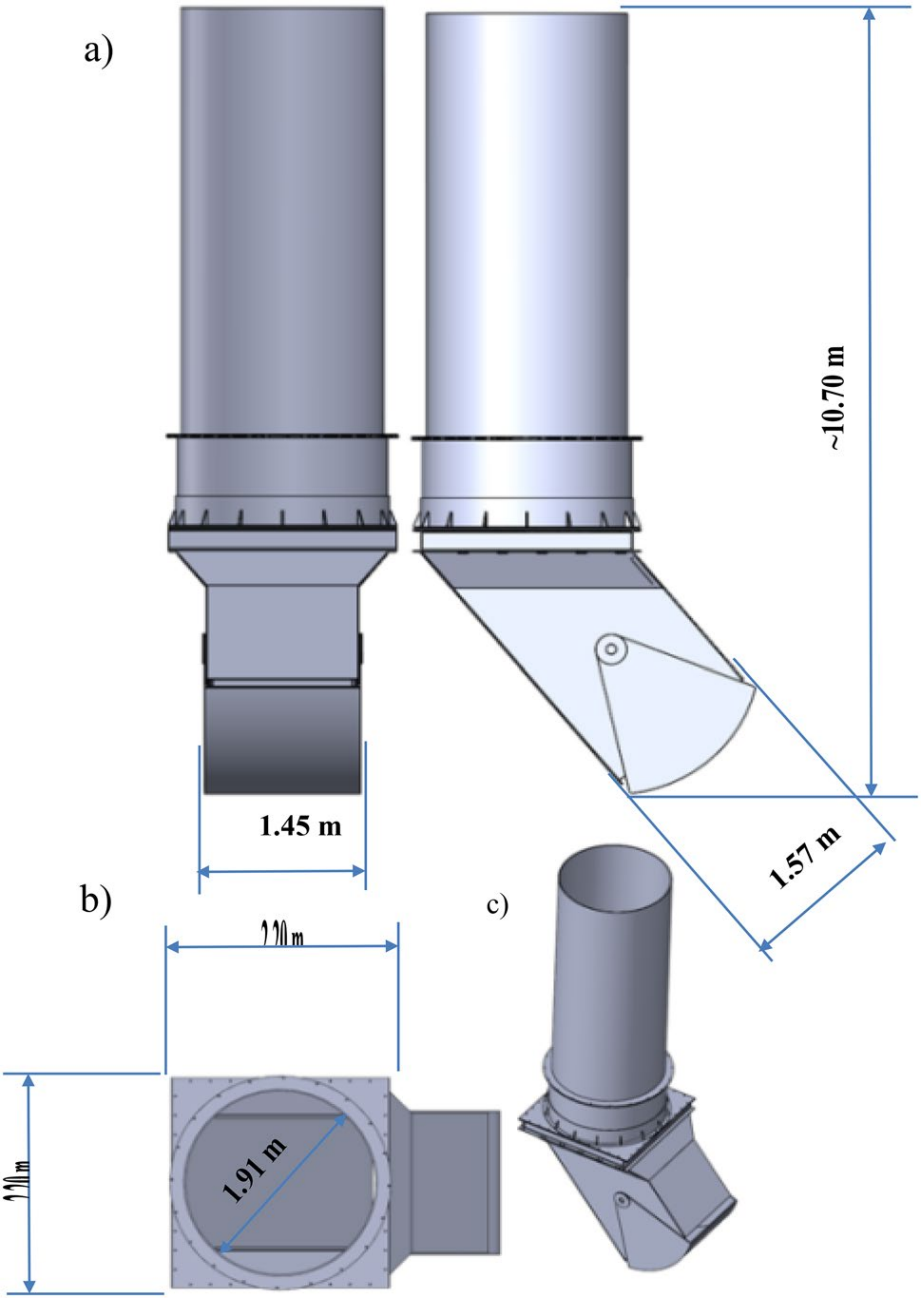
3D model of the Retention bunker: (**a**)—front view, (**b**)—top view, (**c**)—isometric view.

Figure 6 depicts a 3D model of the retention bunker. The underground coal storage bunker has a vertical cylindrical shape with a height of approximately 8.1 m and a diameter of about 1.91 m. The hopper bottom, as shown in Fig. 2-1 and 2, features a square cross-section with dimensions of approximately 3.0 m in height and approximately 1.45 m by 1.6 m in width. The total height of the retention bunker is approximately 10.70 m.

**Figure 7:**
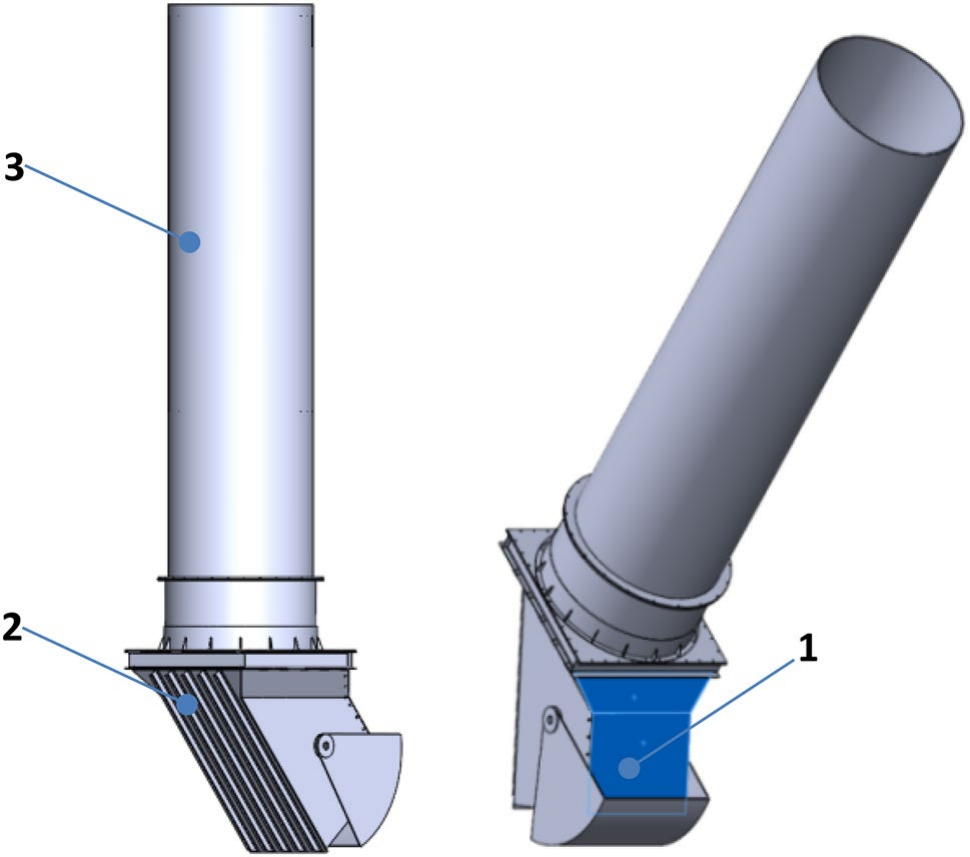
Monitored goals (blue colour) of the underground retention bunker: 1—damaged upper shield, 2—lower shield, 3—tube.

Figure 7 illustrates the location of the damaged element on the retention bunker model, highlighted in blue. This marked element was subjected to monitoring for changes in pressure and force distribution values resulting from static pressure caused by a flowing hydromixture with varying density. The Computational Fluid Dynamics (CFD) model underwent testing for a duration of 3 s.

**Figure 8:**
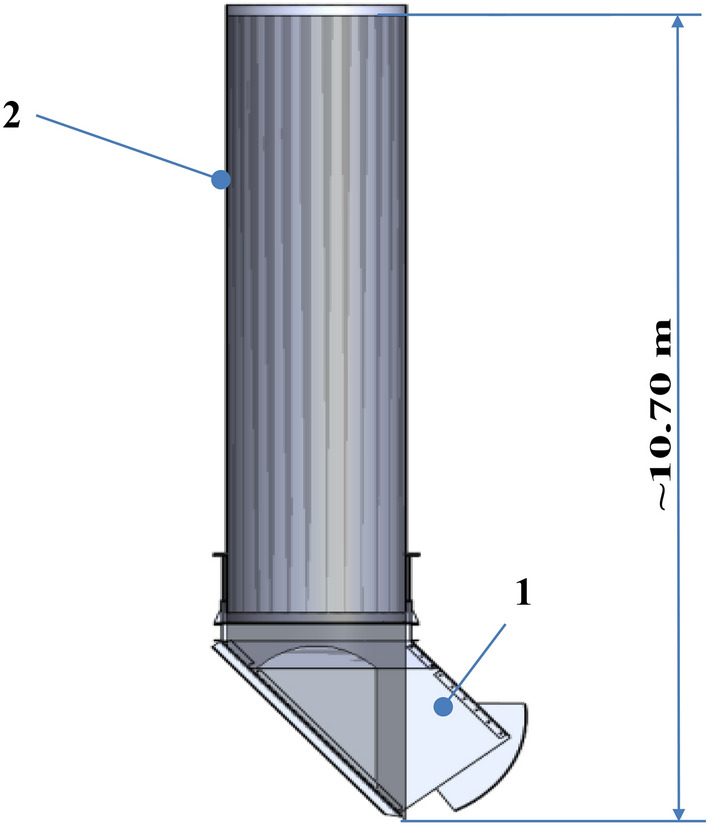
Computational model of the retention bunker (1) and the hydromixture (2) during examination at time t = 0 s.

Figure 8 displays the cross-section of the retention bunker model, highlighting the volume of the hydromixture at time t = 0 s, along with its corresponding dimensions. This visual representation presents the initial distribution and extent of the hydromixture within the bunker.

- Height—10.70 m,
- Diameter—1.91 m,
- Volume—26.63 m^3^.

#### Numerical grid

The choice of mesh type is crucial for accurately simulating fluid flow using the Navier–Stokes equations. In this context, Cartesian meshes were preferred, especially when combined with the Immersed Boundary (IB) method.The Immersed Boundary (IB) technique is particularly advantageous because eliminates the need for a mesh that conforms to boundaries.The process of creating a Cartesian mesh has been started by defining a set of rectangular cells, also known as cuboids or voxels. The cuboids were generated by intersecting planes that are parallel to the axes of the coordinate system.The mesh was improved using a variety of adaptation criteria that can be specified for each solid body (small features, narrow channels, curvature, etc.), as well as automatically in response to gradients in the solution (by splitting each cuboid into 8 geometrically identical cuboids).

**Figure 9:**
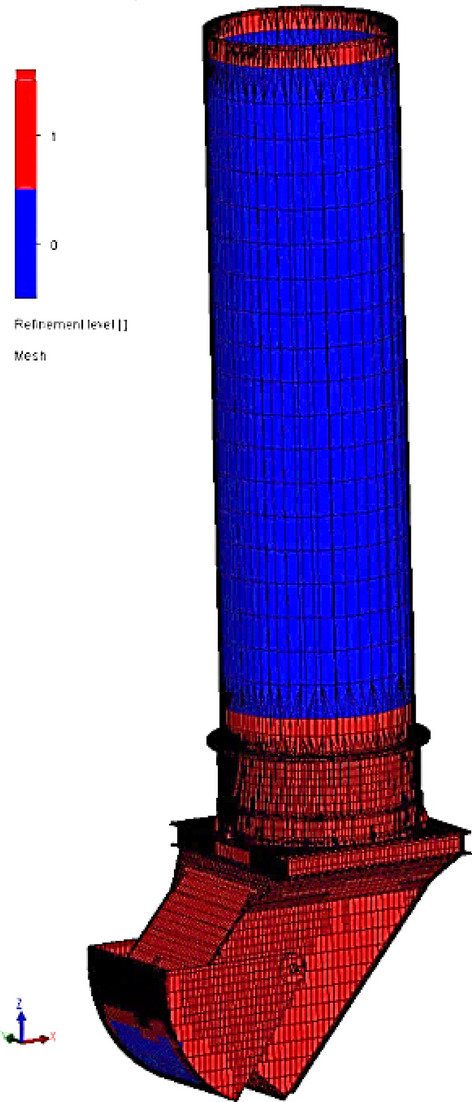
Numerical grid of the retention bunker generated for the purpose of the CFD simulation.

The geometry of the retention bunker provides the basis for creating an internal fluid volume suitable for numerical simulations. To accurately represent the flow behavior within the bunker, a mesh with varying refinement levels is generated, as depicted in Fig. 9.The geometry of the retention bunker has been provided as the basis for creating an internal fluid volume. To accurately represent the flow behavior within the bunker, a mesh with varying refinement levels was generated, as depicted in Fig. 9.

**Figure 10:**
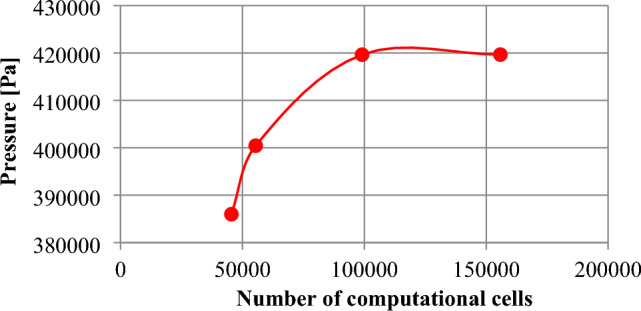
Mesh study results in the numerical grid refinement.

It is important to use an appropriately numerical grid for the purpose to ensure that the results of the CFD numerical calculations are appropriate. Figure 10 depicts a grid refinement study that has been conducted to assess the effect of grid resolution on the results of the inner pressure change due to dynamic interactions with the coal-water mixture and the retention bunker model.The mesh study has been focused on evaluating the numerical grid’s influence on the simulation results, particularly considering a density of 2000 kg/m^3^ for the coal-water mixture.

The results of the mesh study were compared in Table 1.

**Table 1:** Numerical grid refinement study.

No	Mesh quality	Number of computational cells	Pressure [Pa]
1	Coarse mesh	45,455	385,988
2	Normal mesh	55,428	400,440
3	Fine mesh	99,055	419,635
4	Very fine mesh	155,799	419,646

Table 1 shows that the fine and very fine meshes predicted similar results, while the coarse and normal meshes forecast pressure with less accuracy. Based on the results of the convergence investigation, it was decided that the numerical grid in the model would have more than 155,799 computational cells.

**Figure 11:**
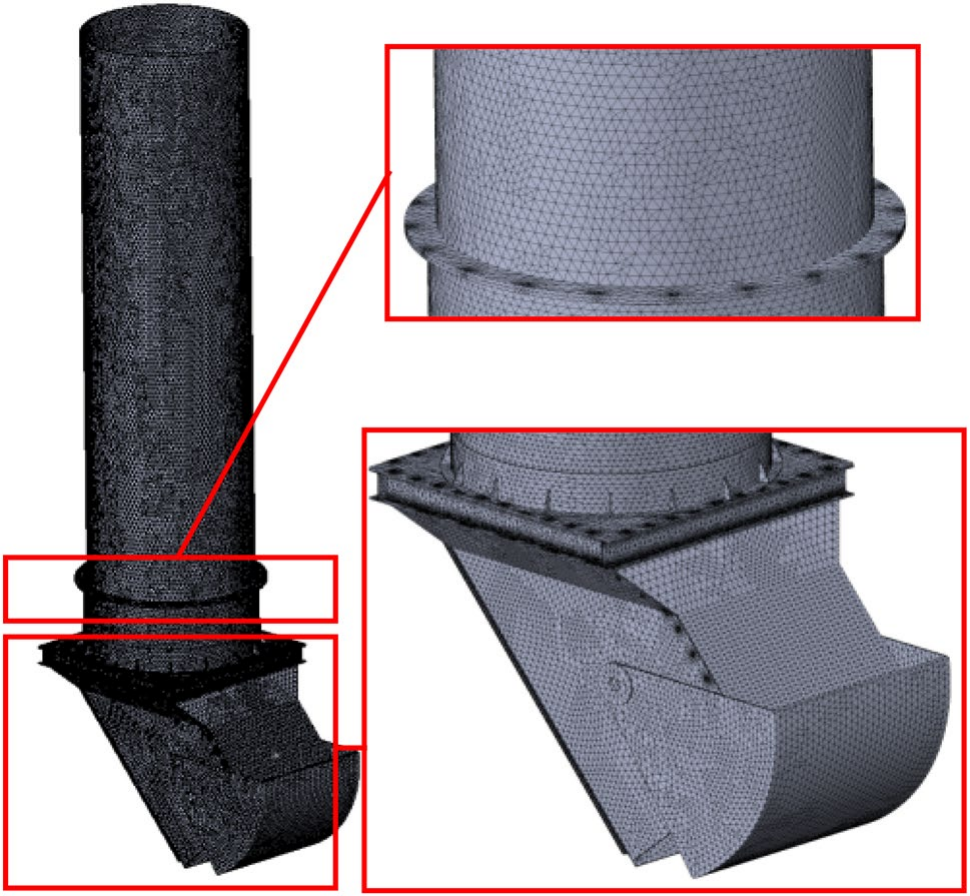
Numerical grid generated for the purpose of the FEM simulation.

A very important stage of the Finite Element Method (FEM) analysis is meshing. The information gathered from each element that makes up the 3D model is combined by FEM to predict the behaviour of the model shown in Fig. 8. The software calculates the model’s global element size while taking its volume, surface area, and other geometric characteristics into account. The geometry and dimensions of the model, the size of the element, the mesh tolerance, the mesh control, and the contact criteria all affect how big the created mesh (number of nodes and elements) will be. Figure 11 shows the numerical grid in form of the 3D tetrahedral solid elements.

The numerical grid shown in Fig. 11 was formed by 501,906 elements, connected with 1,013,521 nodes.

### Informed consent statement

Informed consent was obtained from all subjects involved in the study.

## Results

**Figure 12:**
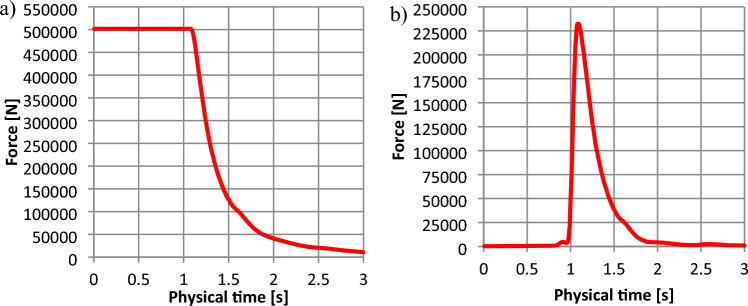
The force distribution depending on the time for the 1450 kg m^−3^: (**a**)—force distribution along the lower shield (Fig. 8-2); (**b**)—force along the upper shield (Fig. 8-1).

**Figure 13:**
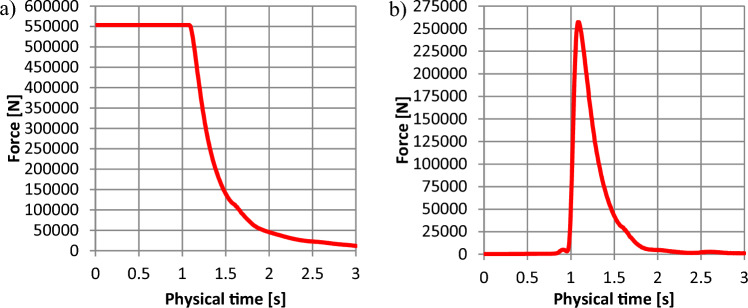
The force distribution depending on the time for the 1600 kg m^−3^: (**a**)—force distribution along the lower shield (Fig. 8-2); (**b**)—force along the upper shield (Fig. 8-1).

**Figure 14:**
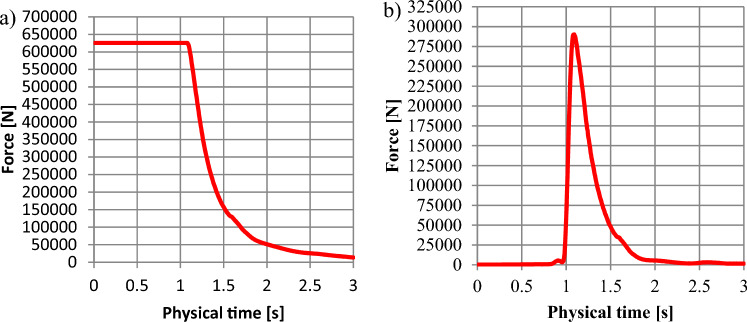
The force distribution depending on the time for the 1800 kg m^−3^: (**a**)—force along the lower shield (Fig. 8-2); (**b**)—force along the upper (damaged) shield (Fig. 8-1).

**Figure 15:**
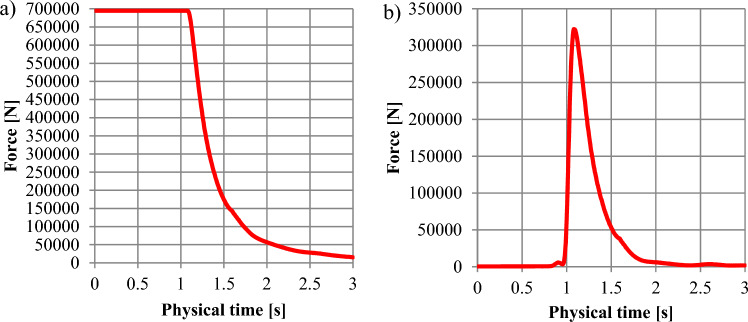
The force distribution depending on the time for the 2000 kg m^−3^: (**a**)—force along the lower shield (Fig. 8-2); (**b**)—force distribution along the upper (damaged) shield (Fig. 8-1).

The results obtained from CFD simulation are shown in Figs. 12–15 and in Table 2. The results obtained from the FEM analysis are shown in Table 3. Figures 12–15 present the force changes at the monitored element of shields in the underground bunker 3D model. Figures 12a–15a present the force distribution in the lower shield of the bunker are shown in Figs. 2-2 and 7-1 Table 2 shows the static pressure changes along the underground bunker for the developed 3D model shown in Fig. 8. The values obtained from the CFD simulation in Table 2 were compared with the results obtained from the mathematical model.

**Table 2:** Comparison of the pressure changes obtained from the mathematical and the CFD model.

Density [kg m^−3^]	Pressure [Pa]		
	Mathematical model	CFD model	
1450	234,571	245,307	
1600	258,837	260,093	
1800	291,192	280,924	
2000	320,494	299,897

**Table 3:** Results of the displacement and the stress distribution obtained from the FEM analysis.

Density [kg m^−3^]	Displacement [m]		H-M-H stress [N m^−2^]
1450	0.0370		
1600	0.0408		
1800	0.0458		
2000	0.0510		

Figure 12 shows that for the hydromixture density of 1450 kg m^−3^, force distribution along the bottom shield (Figs. 2-1 and 7-1) is 501.884 kN for the time interval up to 1.0 s (Fig. 12a), while the force monitored at the upper (damaged) shield (shown in Fig. 2-2 and 7-1) is increasing to a value of about 231.312 kN, reaching its maximum in 1.08 s.

Figure 13 shows that for the hydromixture density of 1600 kg m^−3^, force distribution along the bottom shield (Figs. 2-1 and 7-2) is 553.825 kN for the time interval up to 1.03 s (Fig. 13a), while the force monitored at the upper (damaged) shield (shown in Figs. 2-2, 7-2) is increasing to a value of about 254.846 kN, reaching its maximum in 1.08 s.

Figure 14 shows that for the hydromixture density of 1800 kg m^−3^, force distribution along the bottom shield (Figs. 2-1,7-2) is 625.487 kN for the time interval up to 1.02 s (Fig. 14a), while the force monitored at the upper (damaged) shield (shown in Fig. 2-2,7-2) is increasing to a value of about 288.651 kN, reaching its maximum in 1.08 s.

Figure 15 shows that for the hydromixture density of 2000 kg m^−3^, force distribution along the bottom shield (Figs. 2-1,7-2) is 694.491 kN for the time interval up to 1.075 s (Fig. 15a), while the force monitored at the upper (damaged) shield (shown in Figs. 2-2,7-2) is increasing to a value of about 314.690 kN, reaching its maximum in 1.07 s.

In Figs. 12–15, between 1.0 and 1.5 s, a maximal force peak of about 231 kN, 254 kN, 288 kN, and 314 kN was observed, which caused damage to the bunker shield (Figs 7-1,1-4). This phenomenon is well illustrated in Fig. 16.

**Figure 16:**
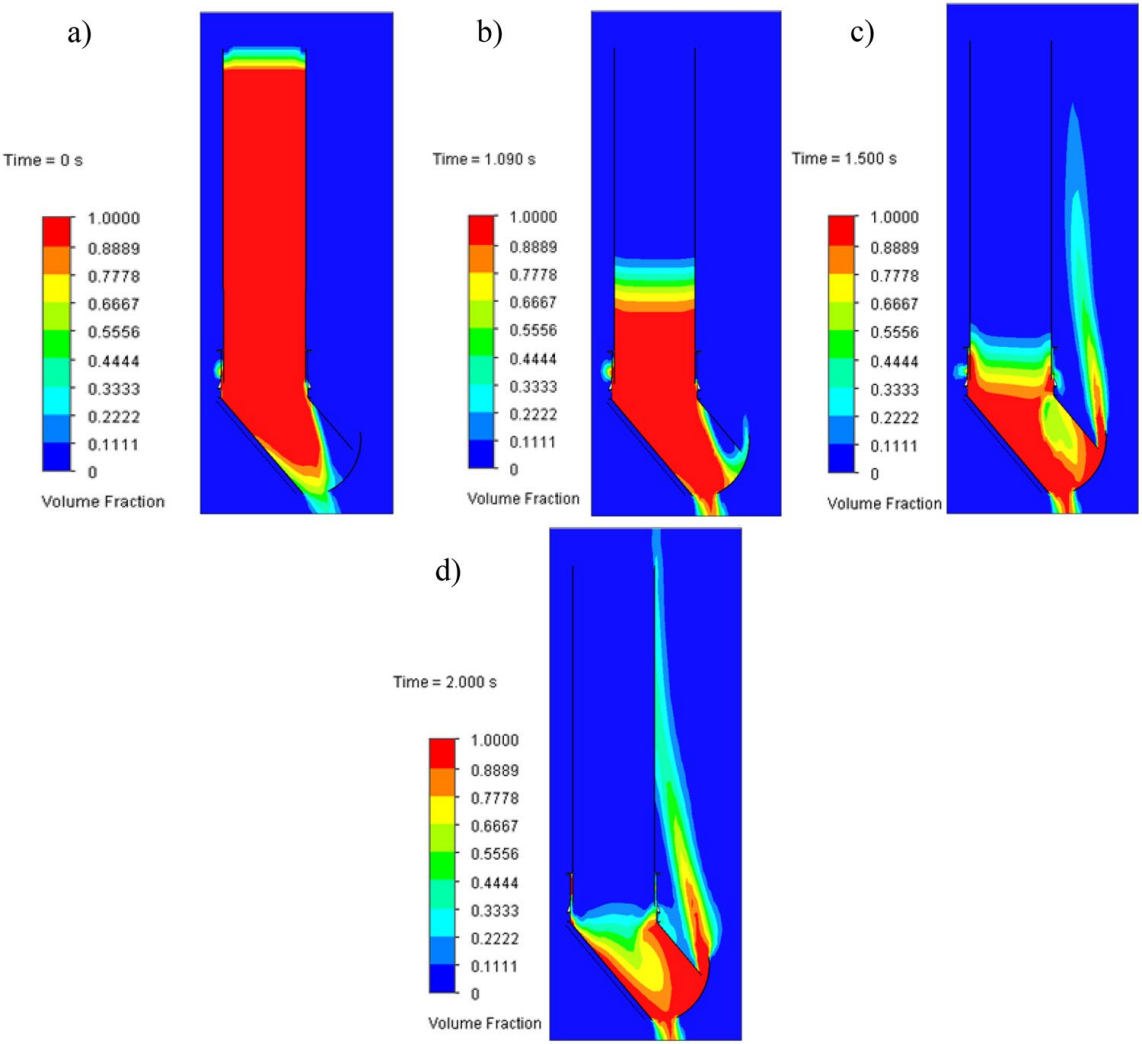
Map of the volume fraction behaviour of the hydromixture during time: (**a**)—for t = 0 s, (**b**)—for t = 1.09 s, (**c**)—for t = 1.5 s and (**d**)—for t = 2.0 s.

The maps in Fig. 16 show that the hydromixture in a time of 1.09 s moves towards the analysed shield of the bunker (presented in Fig. 7-1), reaching full contact after a time of 1.25 s.

The comparison of the pressure change along the underground bunker obtained from the CFD solver and the mathematical models (1) and (2) are presented in Table 2. However, Table 3 presents the results of the displacement and the von Mises stress distribution obtained from the FEM numerical calculations.

**Figure 17:**
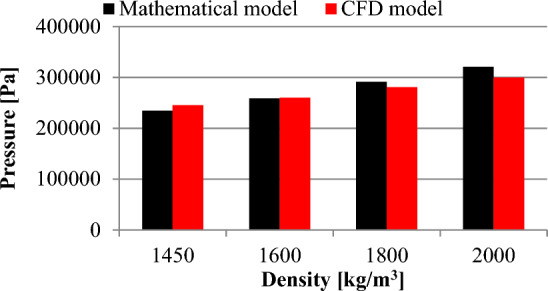
Comparison of the static pressure results obtained from the mathematical model and the CFD numerical simulations.

Table 2 provides a comparison of the pressure values obtained from the mathematical model and the Computational Fluid Dynamics (CFD) numerical simulation for a hydromixture density of 2000 kg m^−3^. The comparison reveals that there is a discrepancy between the pressure values obtained from the mathematical model and the CFD numerical simulation. The pressure value from the mathematical model is higher (320.494 kPa) compared to that from the CFD numerical simulation (299.897 kPa). Similar to the comparison for the hydromixture density of 2000 kg m^−3^, there is a difference between the pressure values obtained from the mathematical model and the CFD numerical simulation for a hydromixture density of 1800 kg m^−3^. In this case, the pressure value from the mathematical model is higher (291.192 kPa) compared to that from the CFD numerical simulation (280.924 kPa). For the hydromixture density of 1600 kg m^−3^, the value of the pressure is 258.837 kPa obtained from the mathematical model, while from the CFD numerical simulation is 260.093 kPa. In the case of the hydromixture density of 1450 kg m^−3^, the value of the pressure is 234.571 kPa obtained from the mathematical model, while from the CFD numerical simulation is 245.307 kPa. The values collected in Table 2 are illustrated in Fig. 17.

The stress values for the analyzed upper shield (Fig. 7-1) in Table 3 exceed the allowable value of 235 MPa. It means that the upper shield has experienced excessive loading, which has led to stress concentrations resulting in the failure of the screw connections.

### Results analysis

**Figure 18:**
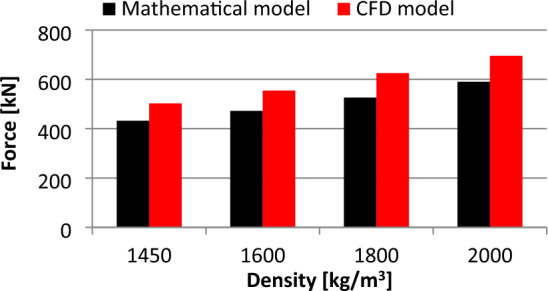
Comparison of the force distribution results obtained from the mathematical model and the CFD numerical simulations.

To estimate the differences in indication error between the pressure values shown in Tables 2 and 3, the following equation can be used^12^:

13 $${\delta }_{x}=\frac{{x}_{numer-{X}_{math}}}{{X}_{math}}\cdot 100\%$$

where: ${x}_{numer}$ is the results obtained from the CFD numerical simulations, ${x}_{math}$ is the results obtained from the mathematical model,

Figure 18 shows the difference in indication error for different hydromixture densities:

- For the hydromixture density of 1450 kg m^−3^, the indication error is approximately 14%.
- For the hydromixture density of 1600 kg m^−3^, the indication error is approximately 16%.
- For the hydromixture density of 1800 kg m^−3^, the indication error is approximately 16%.
- For the hydromixture density of 2000 kg m^−3^, the indication error is approximately 15%.

From the observation of the results shown in Table 3, the displacement of the monitored upper shield, as shown in Fig. 7-1, changes from:

- 0.0370 m for the hydromixture with a density of 1450 kg m^−3^,
- 0.0408 m for the hydromixture with a density of 1600 kg m^−3^,
- 0.0458 m for the hydromixture with a density of 1800 kg m^−3^, to
- 0.0510 m for the hydromixture with a density of 2000 kg m^−3^.

In the case of the stress distribution, it can be observed that the stress value exceeded the allowable stress values (Table 3a). The allowable stress value for the monitored shield is 235 MPa. In addition, it was observed that exceedances of the permissible stresses of the screw connection (Fig. 1-1) were exceeded, as shown in Table 3b. The allowable stress value for the screw connection is 640 MPa.

## Discussion

The novelty and significance of the study lie in its exploration of the dynamic interaction between a retention bunker and a coal-water hydromixture. This aspect represents a crucial yet relatively unexplored domain within underground coal mining operations. By investigating how the presence of water alters the behaviour of stored coal within the bunker, the study sheds light on a complex and often overlooked aspect of mining infrastructure.

Traditional analyses of retention bunkers have primarily focused on static conditions, neglecting the dynamic effects induced by the presence of water. However, in real-world mining scenarios, the infiltration of water into underground storage facilities is a common occurrence, leading to the formation of coal-water hydromixtures. Understanding how these mixtures behave and interact with the bunker structure is paramount for ensuring the safety and efficiency of mining operations.

The study utilizes a combined approach of Computational Fluid Dynamics (CFD) and Finite Element Method (FEM) to simulate and analyze the behavior of the retention bunker under varying conditions. This integration of CFD and FEM allows for a comprehensive investigation of both fluid flow dynamics and structural mechanics within the bunker system, providing a holistic understanding of its performance.

The applied research methodology made it possible to determine that the probable causes of damage to the structural elements of the retention bunker. The inflow of process or natural water led to changes in the physical and mechanical parameters of stored material, such as the angle of internal friction and the angle of natural repose. Internal friction characterises the resistance that rock particles of the same body put under shear stress. The resistance of internal friction is due to the existence of cohesion forces and depends on the freedom of movement of the particles in a given body. The internal friction angle for excavated material typically changes within the range of 16–35°^30^. The constant value of the coefficient of internal friction is necessary for the design of transport and auxiliary equipment and the calculation of their energy effectiveness. The coefficient of friction of the bulk material against the walls of the shielding equipment and components of the conveying machinery depends on whether the excavated material is static or in relative motion. The quantity that characterises frictional forces is the coefficient of friction, whether dynamic during motion or static, when the process of sliding the contacting surfaces of different bodies is initiated. The difference between the value of the coefficient of static and dynamic friction depending on the roughness of the contacting surfaces^24^. The inflow of water increasing the sliding between the surfaces of the excavated material grains. Under conditions of high humidity, due to the inflow of water, the angle of natural repose of the excavated material is disturbed. As a result of this phenomenon, the excavated material moved towards the shield shown in Fig. 8-1, which, according to the retention bunker designer, was not considered a potentially loaded element. A sudden increase in the value of the force observed in Fig. 12b, 13b, 14b and 15b occurred and caused to damage to the retention bunker.

The disturbance in the angle of natural repose of the excavated material resulted in the formation of the fluid hammer, as observed in Fig. 16, and an increase in the allowable force, as observed in Figs. 12a–15a. + The retention bunker was damaged due to hydraulic shock generated by a fluid consisting of coal and rocks suspended in water, which led to shear stresses in the screw connection and damaged the flat steel sheathing shown in Fig. 7-1 as a result.

## Conclusions

The article presents the results of modelling studies to determine the causes of damage to a retention bunker designed to store coal. Model studies were conducted by coupling CFD and FEM methods. CFD numerical simulations made it possible to determine the predicted values of pressure and forces in the retention bunker under the hydromixture flow conditions. The FEM numerical simulations made it possible to determine the predicted changes in stress values and displacement based on the CFD solver results. It was assumed that process water may have flowed into the stored material in the retention bunker, causing a change in the operation condition from static to dynamic. The effect of changes in the density of the hydromixture on the value of pressure changes and stresses in the structural elements of the retention bunker was investigated.

The results of the model tests enabled the formulation of the following conclusions:

- The retention bunker damage was caused by the increase in the values of the force shown in Figs. 12–15, monitored at the retention bunker steel sheathing element presented in Fig. 7-1, due to hydraulic shock caused by the fluid motion consisting of coal and rock suspended in water,
- The loss of stability of the material stored in the retention bunker was caused by the disturbance of the geotechnical parameters of the excavated material due to the inflow of natural or process water,
- The change in density of the hydromixture directly influences the value of shear stress (pressure), displacement, and stresses in the structural elements of the retention bunker,
- The results of the CFD numerical simulation are comparable with the results obtained from the mathematical models, which prove the correctness of the CFD numerical model assumptions adopted,
- The discrepancies between numerical and mathematical results could arise due to various factors, including simplifications or assumptions made in the mathematical model, numerical discretization errors in the CFD simulation, convergence criteria, turbulence modeling, boundary conditions, or other modeling considerations,
- The novelty and significance of the study lie in its exploration of the dynamic interaction between a retention bunker and a coal-water hydromixture. This aspect represents a crucial yet relatively unexplored domain within underground coal mining operations. By investigating how the presence of water alters the behavior of stored coal within the bunker, the study sheds light on a complex and often overlooked aspect of mining infrastructure,
- The use of computational fluid dynamics (CFD) methods in combination with finite element methods (FEM) enables the identification of the pressure (shear stresses) values of hydromixture and the von Mises stress in the shield of the retention bunker, which has contributed to damage as a result of dynamic loads originating from the hydromixture motion,
- The coupling of CFD with FEM provides a synergistic approach, where the fluid flow results from CFD simulations serve as input conditions for the structural analysis conducted with FEM. This integrated simulation framework enables a comprehensive evaluation of the bunker’s performance, considering both fluid–structure interaction effects and their implications on safety, stability, and operational efficiency,
- The utilization of CFD coupled with FEM offers a powerful methodology for simulating and analyzing the behavior of retention bunkers under varying conditions. It enables engineers to gain valuable insights into the complex interplay between fluid dynamics and structural mechanics, guiding the design, optimization, and maintenance of underground coal storage facilities,
- The comparison between the stress values obtained from the numerical analysis and the shield’s designed strength provides valuable insight into the structural integrity and safety of the retention bunker under the analyzed conditions,
- The study showcases the effectiveness of CFD-FEM coupling in predicting the structural response of the retention bunker to dynamic loading from the hydromixture. By integrating CFD simulations with FEM analysis, the study provides insights into the mechanical integrity and stability of the bunker under transient flow conditions.

## Data Availability

All data generated or analysed during this study are included in this published article.
